# Home Advantage and Away Disadvantage of Teams in Champions League: Is It Valid for All Teams and Against Every Opponent?

**DOI:** 10.5114/jhk/175398

**Published:** 2024-02-17

**Authors:** Ümit Kuvvetli, Özgül Vupa Çilengiroğlu

**Affiliations:** 1 Department of Business Administration, Bakircay University, İzmir, Türkiye.; 2 Department of Statistics, Dokuz Eylül University, İzmir, Türkiye.

**Keywords:** : soccer competition, winning, loosing, poisson regression

## Abstract

The home advantage (HA) is a robust phenomenon in soccer whereby the home team wins more games and scores more goals than the away team. Similarly, away disadvantage (AD) means that an away team loses more games or scores less goals than the home team. This study examines the HA and AD values of teams in the UEFA-Champions League, covering the seasons from 2003/2004 to 2021/2022, a total of 2,344 matches. Controlling for team ability differences, the study revealed significant variations in HA, ranging from 32.1% to 79.5%, while AD values ranged from 45.1% to 71.9%. The study further found that HA remained consistent for teams across both the group and knockout stages, while AD varied between these stages. Furthermore, the results suggest that, for certain teams, HA is predominantly manifested against weaker opponents, and the impact of opponent strength on HA and AD is limited.

## Introduction

The phenomenon of home advantage (HA), characterized by the tendency of home teams to win more than half of the games played under a balanced home and away schedule, has attracted considerable attention from researchers (Courneya and Carron, 1992; Ramchandani et al., 2021). Numerous studies have examined the presence of HA in various sports, including basketball (Harris and Roebber, 2019), volleyball (Younghui et al., 2020), handball (Pic, 2018; Volossovitch and Debanne, 2021), women's soccer (Leite and Pollard, 2020), hockey (Arboix-Alió et al., 2020), and athletics (Jamieson, 2010). Also, Pollard et al. (2017) found significant differences between sports, between countries and between sexes in terms of HA by analyzing 15 sports in 65 countries worldwide. The results of that study suggest that the pace of a sport and the dimensions of the playing area have an effect on HA (Pollard et al., 2017).

When examined on a team basis, HA can be defined as the superior performance of home teams in matches played at their own venues (winning more points, scoring more goals, etc.) compared to their opponents. Numerous studies conducted worldwide at the team level (Armatas and Pollard, 2014; Goumas, 2017; Gryko et al., 2020; Marek and Vavra, 2017; Pollard and Gomez, 2009) demonstrate that the vast majority of home teams benefit from this advantage. While home teams having the advantage might imply that away teams are disadvantaged, the situation is not as straightforward as it appears. Similarly to the HA, it is possible to define the away disadvantage (AD) as the performance exhibited by teams in matches played away from their home venues (Goumas, 2017).

Soccer has emerged as the most popular sport globally, attracting billions of spectators. Consequently, a significant number of studies have examined HA in soccer, along with numerous other soccer-related investigations. Much of the research on HA in soccer has focused on specific leagues. For instance, Pollard and Gomez (2009) conducted a study on Southwest European countries, estimating HA values of 69.9% for Spain, 66.9% for France, 65.8% for Portugal, and 65.2% for Italy. In recent years, Ramchandani et al. (2021) reported HA values ranging from 58% to 61% for professional soccer leagues in England. In their study, Pollard and Gomez (2014b) investigated the home advantage in 157 national domestic soccer leagues worldwide, analyzing matches between 2006 and 2012. They found that HA, calculated by comparing the points accrued by home teams to the total points gathered in the league, was present across all continents. However, significant differences were noted among countries, and it was determined that the league with the highest home advantage was in Nigeria (86.8%). That study revealed that regions such as the Andes, Balkans, West Africa, and Central America exhibited pronounced home advantages, while the Baltic Republics and numerous leagues on the Arabian Peninsula displayed lower levels of HA. Variables such as the FIFA ranking (indicative of crowd support), maximum geographical distance between teams, the majority of teams coming from a single city, teams playing at high altitudes, recent occurrence of civil conflicts, and the corruption perception index were found to account for 43% of the variation in HA across the leagues, after accounting for competitive balance. The remaining portion of the variation was attributed to regional, ethnic, and cultural factors, necessitating further exploration. In another study, Pollard and Gomez (2014a) conducted a comparative analysis of HA in women's and men's soccer leagues. Spanning the years 2004 to 2010, that study, based on the analysis of matches played in 26 European leagues, revealed that in women's leagues, HA (overall average of 54.2%) was lower compared to men's leagues (overall average of 60%). Factors such as differential crowd effects on players and referees and gender disparities, among others, were identified as potential reasons for this difference. Furthermore, Pollard and Gomez (2014a) indicated that as the status of women became more akin to that of men within a country, the difference in HA between women's and men's soccer leagues diminished.

The calculation of HA in previous studies has typically relied on straightforward mathematical procedures. HA is commonly determined by calculating the percentage of games won by teams playing at home out of the total number of decided games. Additionally, HA can be quantified by calculating the percentage of points earned by home teams out of the total points available (Pollard, 1986). This method has been widely employed in research for several years (Pollard and Gómez, 2015). However, it is important to note that this method does not take team ability into consideration, which can have an impact on the calculation of HA (Rooney and Kennedy, 2018). Pollard and Stefani (2021) investigated various methods used to measure the HA in different sports, leagues, and teams, as well as the contributions of various factors influencing the HA.

The conventional approach to calculate HA based on points can be misleading when assessing individual teams. Using the point calculation method, it is difficult to make accurate assessments of HA for specific teams. For instance, if team A defeats team B 4-0 in a home match and 1-0 in an away match, both matches would yield 3 points for team A. Therefore, relying solely on points does not provide an accurate reflection of team A’s HA. However, analyzing the number of goals scored and conceded reveals that team A performed better at home against the same opponent compared to the away match, which aligns with the concept of HA. Point-based HA calculations fail to consider the primary objective of a soccer team, which is scoring goals. Moreover, these calculations do not account for team ability. Theoretically, a team that wins all its matches in the league would have a HA of 50% based on point calculations. Similarly, a team that loses all away games and earns only 1 point in home games would have a HA of 100%. These results render the analysis based on point calculations for individual teams controversial. To address this issue, it is recommended to calculate HA or AD based on the number of goals scored and conceded, rather than points. This approach provides a more insightful perspective on HA and AD. There are several approaches to calculate HA based on goals. One approach, proposed by Clarke and Norman (1995), employs the least squares method considering team power and ability. Another approach, utilized by Marek and Vavra (2017), combines matches played against the same opponents and calculates HA based on the total goals scored and conceded in those matches. The approach used in this study, similarly to Goumas (2017), calculates a team's HA as the ratio of goals scored by that team to the total goals scored in the matches, while taking the ability of both teams involved into account.

In the literature, there has been a confusion between home advantage and home performance, as highlighted by Pollard and Stefani (2021) when calculating team-level home advantage. As Pollard and Stefani (2021) emphasized, home performance must be compared with away performance to ensure its relevance to HA. Pollard and Gómez (2015) also discussed this as a problem in their study. However, in our research, a distinct approach was employed. A dataset comprising only matches played at home was utilized to predict the HA, and likewise, a dataset composed exclusively of away matches was used to predict AD. This methodology effectively prevents any potential interaction between matches played at home and away, thus yielding more accurate and isolated insights into these distinct aspects of team performance.

There are studies in the literature that explore HA in the UEFA Champions League (CL) and the various factors that can influence it. One such a study focuses on investigating whether HA in soccer differs based on specific circumstances such as geographical, climatic, cultural, and economic factors. The study analyzed CL matches played between 2008 and 2016 using linear regression as the analytical method. In this particular study, researchers found that the home team’s winning advantage increased when they played at a higher altitude. This suggests that playing at a higher elevation may provide an additional advantage for the home team in terms of HA. The study conducted by Damme and Stijn (2019) provides insights into how specific circumstances, such as altitude, can influence HA in the CL.

In another study analyzing situational variables and performance indicators of soccer match results in the CL between 2010 and 2020, various statistical tests were employed (Parim et al., 2021). The researchers utilized ANOVA and Tukey HSD tests to examine the differences in performance indicators among teams. The performance indicators of the teams were further investigated using multidimensional scaling and decision trees, which allowed for a more comprehensive analysis of the data. The findings of the study revealed that the performance indicators of the teams varied depending on the quality of their opponents, categorized as weak, balanced or strong. This suggests that teams adapt their performance based on the level of challenge posed by their opponents in the CL matches. The study conducted by Parim et al. (2021) shed light on the relationship between situational variables, performance indicators, and match outcomes in the CL, providing valuable insights into the dynamics of team performance in this prestigious competition.

In previous studies investigating team-specific HA estimates in different leagues and organizations, it has been observed that nearly all teams exhibit a HA (Armatas and Pollard, 2014; Clarke and Norman, 1995). However, those studies did not consider the strength of the opponent when calculating HA for the teams. Furthermore, there are limited studies in the literature that investigate the AD at the team level. This study aimed to address these gaps by calculating both HA and AD for teams participating in the UEFA Champions League. Additionally, this study investigated how these advantages change during the group and knockout stages of the tournament and against opponents of varying strength. The inclusion of these aspects and findings in the study is expected to make significant contributions to the existing literature on HA and AD in soccer. By considering the strength of the opponent and analyzing the dynamics of HA and AD in the CL, this study provides a more comprehensive understanding of the factors influencing team performance in this prestigious competition.

## Methods

### Data

The data used in this study encompass matches from the 2003/2004 to 2021/2022 seasons of the UEFA Champions League. In the CL, all matches, except the final, are played in pairs, with each team hosting one match at their home ground. Consequently, the final match of each season, which takes place at a neutral venue devoid of HA, was excluded from the analysis. Notably, during the 2019–2020 season, all matches between the round of 16 and the final were held in Lisbon due to the impact of the Covid-19 pandemic. Since these matches lacked any HA or AD, they were also excluded from the dataset. Ultimately, a total of 2,344 match data were considered for analysis. To enhance the statistical robustness of team-specific calculations, the study focused on 42 teams that had participated in at least 20 home/away matches. By including a sufficient number of matches for each team, the study aimed to increase the reliability and validity of the findings in relation to HA and AD within the CL context.

In this study, an investigation was conducted to examine potential differences in HA and AD values between the group and knockout stages of the UEFA Champions League. To achieve this, data from 17 teams were used, all of which had participated in at least 10 home/away matches in both stages of the tournament. It is worth noting that the number of teams that played in both the group and knockout stages was limited, leading to the selection of this subset of teams for the analyses.

The study also aimed to examine whether the HA or AD of teams varied depending on the strength of their opponents. To achieve this, all teams were categorized into three groups based on the points they earned in UEFA matches during the relevant season. Among the 32 teams participating in the CL, the top eight teams with the highest points were considered “strong”, the bottom eight teams with the lowest points were considered “weak”, and the remaining teams were regarded as “medium-strong” in terms of strength. Based on this grouping, a comparison was conducted for each group of teams that had played enough matches against their respective opponents. Specifically, the analysis included 17 teams that had played at least 10 matches against opponents from all three strength groups, as well as 27 teams that had played at least 10 matches against “strong” and “medium-strong” teams.

For this study, all match data and team ability rankings were obtained from the official UEFA website. The HA and AD values for each team were calculated using only the data specific to that particular team. Separate datasets were created for different analyses conducted in the study, and all analyses were carried out at the team level. This approach allowed for a team-specific examination of HA and AD, ensuring that the results accurately reflected the performance of each individual team.

### Statistical Analysis

This study estimated the HA and AD for each team based on the percentage of goals scored and conceded in their home and away matches, respectively. To calculate HA, the number of goals scored by a team in their home matches was divided by the total number of goals scored and conceded in those matches. For example, if a team scored 60 goals and conceded 20 goals in their home matches, their unadjusted HA would be calculated as 60/(60 + 20) × 100% = 75%. A HA value greater than 50% indicates superior performance in home matches. Similarly, the AD was estimated as the percentage of goals conceded by a team in their away matches. If a team scored 30 goals and conceded 50 goals in their away matches, the unadjusted AD would be calculated as 50/(30 + 50) × 100% = 62.5%. A higher AD value represents inferior performance in away matches. It should be noted that the HA or AD value of 50% for any team indicates no HA or AD.

This study employed a multivariate regression analysis to account for the confounding effect of team ability on the HA and AD calculations. Crude calculations of HA and AD are influenced by differences in team ability; thus controlling this factor is essential in obtaining more accurate results. A paired design was used in the analysis, where each match contributed two observations: one for the home team and one for the away team. Generalized Estimated Equations (GEE) in IBM SPSS Version 26 (IBM, 2019) were used for the repeated measures regression analysis. Repeated measures analysis is suitable when observations occur in pairs, and the outcome of interest is likely to be correlated within each pair. This study treated the individual matches as the “groups”, and the number of goals scored by each of the two opposing teams constituted the “observations”. Since the outcome of interest, namely the number of goals scored, is a discrete count, Poisson errors were specified for the regression model. Robust estimation of variance was employed, which ensures valid standard errors even if the within-group correlations deviate from the correlation structure specified in the model. Robust variance estimation also prevents underestimation of standard errors when count data exhibit over-dispersion, a phenomenon where observed variation exceeds what would be expected from a Poisson distribution. The modeling strategy used in this study had been previously employed to investigate HA in terms of goals scored and disciplinary sanctions issued by referees in soccer. Goumas (2013) provides a comprehensive description of the modeling approach.

To account for variations in the abilities of home and away teams, UEFA assigns points to European soccer teams based on their previous performance in club competitions. In this study, a linear term representing the number of points assigned to each team in each season of the UEFA Champions League was incorporated into the regression model mentioned earlier. However, unlike Goumas (2017), who used the teams' previous season points, this study used the points ranking of the respective season to which the match belonged. This approach was adopted to reflect the strength and performance of teams in the matches of the respective season more accurately. By including the points ranking of the corresponding season, the regression model took into consideration the varying abilities of teams in different seasons and provided a more realistic assessment of the impact of team ability on HA and AD.

Linear combinations of equations were used to estimate adjusted HA and AD in terms of the percentage of goals scored in home matches by each team (HA) and the percentage of goals conceded in away matches by each team (AD). HA and AD were derived from the Poisson regression coefficient (β) for match location (0 = Away, 1 = Home) for each team using the following equation:


$$HA\;and\;AD=\frac{\exp\left(\beta \right)}{\exp\left(\beta \right)+1}\times 100\%$$

The standard error (SE) for HA and AD can be calculated as follows, where SE is the standard error of beta (Goumas, 2013):


$$SE\left(HA\;and\;AD\right)=HA-\left(\frac{\exp\left(\beta -se\right)}{\exp\left(\beta -se\right)+1}x100\right)$$

In these equations, the exponential function exp (β) is applied to the regression coefficient for the match location, and the results are transformed into percentages. The HA represents the estimated percentage of goals scored by the home team, while the AD represents the estimated percentage of goals conceded by the away team. Using this approach, the study aimed to obtain adjusted estimates of HA and AD that would consider the impact of the match location on goal scoring and conceding, providing a more accurate assessment of the influence of HA and AD in soccer matches in the UEFA CL. To test for variation in HA and AD between teams, a chi-square test was carried out; *p* values less than 0.05 were considered significant.

Detailed information regarding the methodology can be found in the study of Goumas (2017), which serves as a reference for the present research. However, the current study expands upon Goumas' work by calculating HA and AD values for a larger number of teams. Moreover, this study investigated changes in HA and AD values for the same teams during both the group and knockout stages of the competition. Additionally, we examined HA and AD values for each team based on the strength of their opponents. These additional analyses aimed to provide a more comprehensive understanding of HA and AD in the context of the UEFA Champions League.

## Results

### Results for HA

In the analyzed period, a total of 122 different teams participated in the UEFA Champions League. However, only 42 of these teams played at least 40 matches, making them eligible for further analysis. Table 1 provides a summary of the number of home matches, goals scored and conceded, as well as both crude and adjusted HA values for these teams. Additionally, the chi-square *p*-values were included, indicating the statistical significance of the adjusted HA values. The adjusted HA values took into account factors such as team ability, the season, and the stage of competition. These values were utilized to rank the teams in descending order based on their expected level of HA when playing against opponents of equal ability. By adjusting for these factors, the analysis aimed to eliminate any variation between teams that might arise due to confounding effects of the season and the stage of the competition (Goumas, 2017).

**Table 1:** HA (%) for teams in the UEFA CL in 2003/2004 to 2021/2022 seasons.

				Home Advantage (%)		
Team	Home matches	Goals for	Goals against	Crude	Adj (SE)	*p*-value
Sevilla	27	49	34	59.0	79.5 (3.0)	<0.001
Bayern München	92	246	75	76.6	77.7 (1.2)	<0.001
Ajax	41	63	49	56.3	76.2 (3.0)	<0.001
Sporting Lisbon	23	35	39	47.3	76.0 (6.6)	0.001
Borussia Dortmund	42	84	45	65.1	75.8 (2.1)	<0.001
Barcelona	93	236	68	77.6	74.6 (1.4)	<0.001
Tottenham Hotspur	22	44	27	62.0	74.0 (4.7)	<0.001
Paris Saint-Germain	49	111	48	69.8	73.8 (2.3)	<0.001
Real Madrid	97	239	99	70.7	73.8 (1.4)	<0.001
Valencia	32	58	44	56.9	73.7 (5.0)	<0.001
Schalke 04	30	48	41	53.9	73.5 (3.5)	<0.001
Manchester City	50	115	56	67.3	73.1 (2.7)	<0.001
Club Brugge	21	17	40	29.8	72.8 (8.8)	0.016
Galatasaray	27	31	39	44.3	72.5 (7.5)	0.006
Bayer Leverkusen	26	48	38	55.8	72.2 (5.2)	<0.001
Arsenal	63	128	52	71.1	72.1 (3.3)	<0.001
Napoli	21	43	23	65.2	71.7 (5.5)	<0.001
Manchester United	71	137	59	69.9	71.0 (2.4)	<0.001
Dinamo Kiev	34	43	53	44.8	71.0 (5.6)	0.001
AS Monaco	27	43	34	55.8	70.7 (5.9)	0.001
Liverpool	60	119	49	70.8	70.4 (2.8)	<0.001
Shakhtar Donetsk	50	72	65	52.6	70.2 (5.0)	<0.001
Milan	51	82	44	65.1	70.0 (2.7)	<0.001
Porto	69	109	67	61.9	69.7 (3.5)	<0.001
Zenit St.Petersburg	30	43	35	55.1	69.6 (5.4)	0.001
Chelsea	85	168	68	71.2	69.4 (2.2)	<0.001
Roma	38	63	46	57.8	69.3 (4.5)	<0.001
Internazionale	53	79	55	59.0	68.6 (3.2)	<0.001
Benfica	51	63	49	56.3	68.3 (3.1)	<0.001
Olympique Lyon	55	92	57	61.7	68.3 (3.9)	<0.001
Basel	24	28	34	45.2	68.0 (8.4)	0.039
Juventus	67	112	52	68.3	67.8 (2.4)	<0.001
Olympique Marseille	27	32	35	47.8	66.2 (6.2)	0.011
Atlético Madrid	50	78	31	71.6	64.9 (4.1)	<0.001
Werder Bremen	20	37	29	56.1	64.7 (5.8)	0.014
Olympiakos Piraeus	45	61	57	51.7	64.3 (6.0)	0.020
Anderlecht	24	17	47	26.6	62.4 (15.4)	0.418
Celtic	30	32	35	47.8	61.0 (9.6)	0.255
PSV Eindhoven	34	38	33	53.5	60.6 (4.9)	0.032
Villarreal	20	21	22	48.8	60.2 (7.4)	0.168
CSKA Moscow	36	38	49	43.7	56.0 (7.8)	0.438
Lille	20	13	19	40.6	32.1 (11.0)	0.189

Table 1 reveals that out of the 42 teams included in the analysis, 37 teams (excluding Anderlecht, Celtic, Villareal, CSKA Moscow, and Lille) exhibited a significant HA. The HA values ranged from 32.1% to 79.5%, indicating substantial variation among teams (including Lille, $\chi_{41}^{2}=88.4,\;p<0.001$, excluding Lille, $\chi_{40}^{2}=60.1,\;p=0.03).$ These findings contrast with Goumas (2017), who reported no difference in HA among teams that played at least 50 matches in 10 seasons of the UEFA CL. One possible explanation for this discrepancy is that the Goumas' study focused on consistently participating teams who were among the strongest in the league. However, when examining the subset of 20 teams that played at least 40 matches in the UEFA CL, HA values ranged from 64.3% to 77.7%. Similarly to Goumas' findings, there was no significant difference (χ^2^_19_ = 14.8, *p* = 0.73) in HA among these teams. This suggests that there may be a relationship between HA in the UEFA CL and factors such as team strength, participation experience, and country, among others. It is important to note that these results are specific to the teams analyzed in this study and should be interpreted within the context of the data and methodology employed. Further research is warranted to explore the potential factors contributing to variations in HA across teams in the UEFA Champions League.

Table 2 summarizes the adjusted HA values for the overall league, the group stage, and the knock-out phase, as well as the corresponding *p*-values indicating their statistical significance, for the 17 teams that played at least 10 matches in both stages. All teams listed in the table exhibited significant HA values in both the overall league and group stages. Except for A. Madrid, they also demonstrated significant HA values in the knock-out phase. Although HA values of the teams in Table 2 ranged between 67.6% and 78.6% in the group stage, no significant difference was observed among the teams $\left(\chi_{16}^{2}=10.3,\;p=0.85\right)$. Similarly, there was no significant difference among these teams in the knock-out stage $\left(\chi_{16}^{2}=15.1,\;p=0.51\right)$. Furthermore, when comparing HA values of teams between the group stage and the knock-out phase on an individual basis, no significant difference was found (*p* = 0.740), indicating that the teams had similar HA values in both the group and knock-out stages (Table 2).

**Table 2:** HA (%) for teams in the group stage and the knock-out phase in the UEFA Champions League.

	All			group stage			knock-out phase		
Team	HM	Adj (SE)	*p*-value	HM	Adj (SE)	*p*-value	HM	Adj (SE)	*p*-value
Bayern München	92	77.7 (1.2)	<0.001	54	78.6 (1.5)	<0.001	38	75.7 (2.9)	<0.001
Borussia Dortmund	42	75.8 (2.1)	<0.001	30	73.8 (3.4)	<0.001	12	77.7 (4.3)	<0.001
Barcelona	93	74.6 (1.4)	<0.001	54	76.1 (1.6)	<0.001	39	73.4 (2.6)	<0.001
Paris Saint-Germain	49	73.8 (2.3)	<0.001	33	76.6 (2.9)	<0.001	16	66.5 (5.1)	0.002
Real Madrid	97	73.8 (1.4)	<0.001	57	75.4 (1.9)	<0.001	40	73.1 (2.0)	<0.001
Manchester City	50	73.1 (2.7)	<0.001	33	75.4 (2.5)	<0.001	17	71.5 (5.9)	<0.001
Arsenal	63	72.1 (3.3)	<0.001	42	70.0 (4.4)	<0.001	21	76.0 (5.8)	<0.001
Manchester United	71	71.0 (2.4)	<0.001	48	71.2 (3.0)	<0.001	23	73.8 (4.2)	<0.001
Liverpool	60	70.4 (2.8)	<0.001	36	73.9 (3.3)	<0.001	24	66.9 (4.6)	<0.001
Milan	51	70.0 (2.7)	<0.001	33	71.2 (3.3)	<0.001	18	70.6 (3.6)	<0.001
Porto	69	69.7 (3.5)	<0.001	51	70.5 (4.3)	<0.001	18	75.0 (6.0)	<0.001
Chelsea	85	69.4 (2.2)	<0.001	51	68.7 (3.2)	<0.001	34	70.3 (3.1)	<0.001
Roma	38	69.3 (4.5)	<0.001	27	71.5 (5.9)	0.001	11	73.4 (7.4)	0.004
Internazionale	53	68.6 (3.2)	<0.001	39	69.0 (4.2)	<0.001	14	70.2 (2.8)	<0.001
Olympique Lyon	55	68.3 (3.9)	<0.001	39	70.0 (4.0)	<0.001	16	75.7 (7.0)	0.001
Juventus	67	67.8 (2.4)	<0.001	45	67.6 (3.9)	<0.001	22	67.7 (3.4)	<0.001
Atlético Madrid	50	64.9 (4.1)	<0.001	33	71.9 (3.7)	<0.001	17	54.4 (9.2)	0.636

HM: Home matches

Table 3 presents HA values for teams playing against different strength opponents. HA values were calculated for teams that had played at least 10 matches against teams within the same group. Several interesting findings emerge from this analysis. For example, Sporting Lisbon, despite having the highest HA value as the 4^th^ team, did not exhibit any advantage when playing against strong teams at home. Similar observations can be made for many other teams. Furthermore, it can be argued that Sevilla, despite having the highest HA value, obtained this value primarily against medium-strong and weak-level teams since they did not play enough matches against strong teams at home. These findings suggest that HA values may be influenced by the strength of the opponents faced by the teams.

**Table 3:** HA (%) for teams playing against strong, medium-strong and weak teams in the UEFA Champions League.

	all		against “strong” teams		against “medium-strong” teams		against “weak” teams	
Team	Adj (SE)	*p*-value	Adj (SE)	*p-*value	Adj (SE)	*p*-value	Adj (SE)	*p-*value
Sevilla	79.5 (3.0)	<0.001	n/a		71.1 (10.5)	0.057	n/a	
Bayern München	77.7 (1.2)	<0.001	74.8 (3.8)	<0.001	78.8 (2.3)	<0.001	77.8 (3.6)	<0.001
Ajax	76.2 (3.0)	<0.001	65.9 (6.8)	0.023	76.0 (6.4)	<0.001	74.1 (3.4)	<0.001
Sporting Lisbon	76.0 (6.6)	0.001	45.7 (21.6)	0.861	n/a		n/a	
Borussia Dortmund	75.8 (2.1)	<0.001	69.1 (6.5)	0.006	77.5 (3.3)	<0.001	n/a	
Barcelona	74.6 (1.4)	<0.001	69.7 (3.8)	<0.001	76.2 (2.4)	<0.001	77.4 (3.2)	<0.001
Tottenham Hotspur	74.0 (4.7)	<0.001	n/a		n/a		n/a	
Paris Saint-Germain	73.8 (2.3)	<0.001	68.8 (6.2)	0.004	67.7 (3.5)	<0.001	68.1 (11.0)	0.110
Real Madrid	73.8 (1.4)	<0.001	69.8 (2.9)	<0.001	73.5 (1.8)	<0.001	79.6 (3.2)	<0.001
Valencia	73.7 (5.0)	<0.001	36.9 (12.1)	0.350	76.5 (7.1)	0.001	n/a	
Schalke 04	73.5 (3.5)	<0.001	56.2 (20.0)	0.758	74.3 (4.7)	<0.001	n/a	
Manchester City	73.1 (2.7)	<0.001	69.6 (5.3)	0.001	72.8 (4.8)	<0.001	77.8 (3.2)	<0.001
Club Brugge	72.8 (8.8)	0.016	n/a		27.1 (15.6)	0.346	n/a	
Galatasaray	72.5 (7.5)	0.006	n/a		69.5 (8.3)	0.026	n/a	
Bayer Leverkusen	72.2 (5.2)	<0.001	46.7 (23.4)	0.900	n/a		n/a	
Arsenal	72.1 (3.3)	<0.001	68.7 (6.6)	0.007	66.0 (9.4)	0.096	73.2 (5.5)	<0.001
Napoli	71.7 (5.5)	<0.001	n/a		65.5 (14.3)	0.280	n/a	
Manchester United	71.0 (2.4)	<0.001	68.4 (6.3)	0.006	70.1 (3.9)	<0.001	71.8 (6.3)	0.001
Dinamo Kiev	71.0 (5.6)	0.001	61.9 (8.9)	0.184	63.6 (12.3)	0.272	n/a	
Monaco	70.7 (5.9)	0.001	n/a				n/a	
Liverpool	70.4 (2.8)	<0.001	72.0 (4.4)	<0.001	69.6 (5.6)	0.001	72.2 (4.3)	<0.001
Shakhtar Donetsk	70.2 (5.0)	<0.001	65.0 (10.1)	0.143	57.8 (12.1)	0.518	79.1 (5.3)	<0.001
Milan	70.0 (2.7)	<0.001	71.2 (4.0)	<0.001	65.7 (6.0)	0.011	n/a	
Porto	69.7 (3.5)	<0.001	59.1 (7.6)	0.229	71.0 (6.0)	0.001	79.9 (5.4)	<0.001
Zenit St. Petersburg	69.6 (5.4)	0.001	n/a		71.0 (6.4)	0.002	n/a	
Chelsea	69.4 (2.2)	<0.001	67.0 (4.1)	<0.001	67.0 (4.1)	<0.001	74.1 (5.5)	<0.001
Roma	69.3 (4.5)	<0.001	69.5 (8.9)	0.036	75.0 (7.9)	0.005	n/a	
Internazionale	68.6 (3.2)	<0.001	55.7 (12.4)	0.649	67.5 (5.6)	0.003	72.8 (4.7)	<0.001
Benfica	68.3 (3.1)	<0.001	44.0 (9.9)	0.562	73.4 (4.5)	<0.001	n/a	
Olympique Lyon	68.3 (3.9)	<0.001	53.4 (9.7)	0.728	77.1 (5.6)	<0.001	78.2 (5.2)	<0.001
FC Basel	68.0 (8.4)	0.039	n/a		62.8 (10.9)	0.243	n/a	
Juventus	67.8 (2.4)	<0.001	61.5 (5.5)	0.041	59.9 (5.6)	0.082	70.4 (5.7)	0.001
Olympique Marseille	66.2 (6.2)	0.011	56.1 (11.4)	0.595	42.1 (14.6)	0.627	n/a	
Atlético Madrid	64.9 (4.1)	<0.001	59.4 (6.7)	0.162	69.5 (5.9)	0.002	78.2 (5.8)	<0.001
Werder Bremen	64.7 (5.8)	0.014	n/a		59.3 (9.3)	0.319	n/a	
Olimpiakos Piraeus	64.3 (6.0)	0.020	63.7 (16.6)	0.408	53.0 (11.1)	0.789	69.0 (16.9)	0.264
Anderlecht	62.4 (15.4)	0.418	n/a		53.7 (19.2)	0.849	n/a	
Celtic	61.0 (9.6)	0.255	68.0 (31.4)	0.563	76.4 (12.5)	0.052	n/a	
PSV Eindhoven	60.6 (4.9)	0.032	56.0 (12.1)	0.620	49.3 (7.7)	0.930	n/a	
Villarreal	60.2 (7.4)	0.168	n/a		67.6 (8.5)	0.045	n/a	
CSKA Moscow	56.0 (7.8)	0.438	n/a		51.8 (12.7)	0.885	n/a	
Lille	32.1 (11.0)	0.189	n/a		37.9 (16.3)	0.535	n/a	

n/a: not applicable

The analysis of individual teams’ HA values revealed significant differences among the teams. The findings from Table 1 indicate that there was a notable disparity in HA when comparing teams playing against “strong” opponents (χ^2^ = 98.9, *p* < 0.001) and “medium-strong” opponents (χ^2^ = 155.2, *p* < 0.001). However, no significant difference in HA was found against “weak” opponents (χ^2^ = 8.2, *p* = 0.94) in the league. These results suggest that teams with a certain number of matches and experience in the UEFA Champions League generally exhibit HA when playing against “weak” opponents in their home matches. However, the situation becomes more complex when facing “strong” and “medium-strong” teams, as HA is not consistently observed in all teams. It is worth noting that the absence of a significant difference in HA in teams playing against “weak” opponents could be attributed to various factors, such as the teams' familiarity with the CL environment and the overall quality of the competition. Further investigation is required to better understand the dynamics behind the varying HA values among teams when facing opponents of different strengths in the UEFA CL.

When analyzing the team-based HA values, significant and meaningful results were obtained. Among the 17 teams examined, it was observed that their HA values varied significantly (*p* < 0.001) depending on the strength of the opponent they faced. For these 17 teams, HA values did not show significant variation (*p* = 0.391) when playing against “strong” and “medium-strong” opponents. However, it was found that the same teams' HA values differed significantly (*p* < 0.001) when facing “strong” and “weak” opponents, as well as “medium-strong” and “weak” opponents (*p* = 0.002). These findings suggest that these specific teams tended to have a higher HA value when competing against “weak” opponents. The difference in HA against “strong” and “weak” opponents, as well as “medium-strong” and “weak” opponents, indicates that these teams were more likely to perform better in their home matches when facing weaker opponents compared to stronger or medium-strong opponents.

The analysis focused on 42 teams that had participated in the UEFA Champions League (CL) and played a minimum of 40 matches. However, due to the elimination of “weak” teams after the group stage, many of these teams had limited opportunities to face such opponents. As a result, the comparison was primarily conducted among the “strong” and “medium-strong” teams in the league. By excluding the “weak” teams from the analysis, it was possible to identify 27 teams that had played enough matches (at least 10 matches) against the “strong” and “medium-strong” teams. The HA values of these 27 teams were examined and compared. The findings indicated that there were significant variations in the HA of the teams depending on the strength of their opponents (*p* = 0.035). In other words, the advantages teams enjoyed in their home matches differed based on the strength of the opposing teams in the UEFA CL.

### Results for AD

Table 4 presents the AD values for the 42 teams, listed in ascending order based on their AD. The AD values for these teams ranged from 45.1% to 71.9%. Similarly to the findings for HA, there was a significant difference $\left(\chi_{41}^{2}=179.8,\;p<0.001\right)$ among the teams regarding their AD in the UEFA Champions League. Among the 42 teams, 12 teams experienced AD in their CL matches, while the remaining 30 teams did not exhibit a significant AD. This indicates that, for a considerable number of teams, there was no clear pattern of underperformance in their away matches, suggesting their ability to maintain a relatively balanced performance regardless of the match location. These findings highlight the varying performance levels and tendencies of teams in away matches, as reflected in the AD values. Further analysis and investigation into the factors influencing the AD values can provide valuable insights into the dynamics of team performance in different match settings within the UEFA Champions League context.

**Table 4:** AD (%) for teams in the UEFA Champions League in 2003/2004 to 2021/2022 seasons.

				Away Disadvantage (%)		
Team	Away matches	Goals for	Goals against	Crude	Adj (SE)	*p*-value
Paris Saint-Germain	49	91	62	40.5	45.1 (3.8)	0.196
Manchester City	50	90	64	41.6	45.2 (3.8)	0.207
Real Madrid	97	173	113	39.5	46.4 (3.3)	0.276
Manchester United	71	90	70	43.8	46.5 (4.0)	0.378
Chelsea	85	129	88	40.6	46.7 (3.8)	0.379
Liverpool	60	102	65	38.9	46.9 (4.8)	0.516
Juventus	67	85	65	43.3	47.2 (4.0)	0.479
Bayern München	92	173	111	39.1	48.7 (3.2)	0.678
Ajax	41	57	61	51.7	50.7 (4.5)	0.866
Barcelona	93	134	96	41.7	51.6 (3.9)	0.687
Tottenham Hotspur	22	37	38	50.7	52.0 (4.9)	0.684
Sevilla	27	33	35	51.5	52.3 (6.4)	0.724
Olympique Lyon	55	81	76	48.4	52.5 (4.6)	0.956
Internazionale	53	63	68	51.9	52.5 (4.3)	0.559
Valencia	32	33	36	52.2	53.4 (5.8)	0.564
Arsenal	63	84	90	51.7	54.4 (4.2)	0.299
Porto	69	84	97	53.6	54.4 (3.2)	0.178
Borussia Dortmund	42	62	71	53.4	54.9 (3.8)	0.211
Villarreal	20	22	28	56.0	56.3 (6.7)	0.369
Milan	51	56	62	52.5	56.3 (5.2)	0.242
Anderlecht	24	17	48	73.8	56.3 (8.8)	0.495
Club Brugge	21	20	34	63.0	56.7 (7.9)	0.417
Napoli	21	25	35	58.3	56.9 (4.4)	0.128
Sporting Lisbon	23	25	41	62.1	57.5 (5.9)	0.218
Atlético Madrid	50	51	55	51.9	58.9 (4.9)	0.080
Monaco	27	31	44	58.7	59.6 (5.9)	0.126
Schalke 04	30	34	47	58.0	59.8 (5.8)	0.112
Olympique Marseille	27	27	44	62.0	59.8 (7.0)	0.186
Lille	20	18	31	63.3	60.3 (7.1)	0.175
CSKA Moscow	36	42	71	62.8	60.4 (4.6)	0.033
Dinamo Kiev	34	24	58	70.7	61.2 (6.1)	0.086
Bayer Leverkusen	26	23	43	65.2	61.9 (5.6)	0.047
Shakhtar Donetsk	50	58	102	63.8	62.0 (4.6)	0.015
Roma	38	48	82	63.1	63.1 (4.1)	0.003
Basel	24	23	51	68.9	64.0 (5.4)	0.018
Zenit St.Petersburg	30	25	51	67.1	65.1 (5.3)	0.011
Benfica	51	49	92	65.2	65.7 (3.9)	<0.001
Olympiakos	45	38	92	70.8	66.5 (4.5)	0.001
Werder Bremen	20	21	42	66.7	69.1 (4.4)	<0.001
Galatasaray	27	21	63	75.0	69.6 (5.9)	0.006
Celtic	30	21	70	76.9	71.1 (5.2)	0.001
PSV Eindhoven	34	22	59	72.8	71.9 (4.5)	<0.001

The AD values of the 17 teams that participated in at least 10 matches in both the group and knockout stages were analyzed, and the findings are presented in Table 5. The table reveals that none of the teams experienced AD in the UEFA CL overall. However, significant differences were observed when comparing AD values between the group and knockout stages. For instance, Paris Saint-Germain not only avoided AD, but also demonstrated an advantage in their group matches. In contrast, during the knockout stage, it was found that five teams experienced AD. The differences in AD among the teams were more prominent in the knockout stage, as indicated by significant variations observed in both the group stage $\left(\chi_{16}^{2}=41.5,\;p<0.001\right)$ and the knockout stage $\left(\chi_{16}^{2}=57.1,\;p<0.001\right)$. Furthermore, when comparing AD values of the same teams between the group and knockout stages, significant differences (*p* < 0.001) were identified. This suggests that the success of teams in progressing through the tournament was more closely associated with their AD rather than their HA values, as observed in Tables 2 and 4. These results highlight the dynamic nature of team performance in away matches during different stages of the UEFA Champions League. Changes in AD values emphasize the importance of adapting strategies and tactics to the specific challenges posed by the knockout stage, which may lead to variations in team performance. Further exploration of factors contributing to these AD variations can offer valuable insights into the dynamics of team performance in high-stakes CL matches.

**Table 5:** AD (%) for teams in the group stage and the knock-out phase in the UEFA Champions League.

	All			group stage			knock-out phase		
Team	AM	Adj (SE)	*p*-value	AM	Adj (SE)	*p*-value	AM	Adj (SE)	*p*-value
Paris Saint-Germain	49	45.1 (3.8)	0.196	33	40.6 (4.4)	0.036	16	53.4 (6.9)	0.629
Manchester City	50	45.2 (3.8)	0.207	33	44.7 (4.6)	0.250	17	48.5 (6.6)	0.826
Real Madrid	97	46.4 (3.3)	0.276	57	45.0 (4.5)	0.261	40	54.4 (4.8)	0.366
Manchester United	71	46.5 (4.0)	0.378	48	43.7 (4.7)	0.178	23	54.3 (7.0)	0.549
Chelsea	85	46.7 (3.8)	0.379	51	39.4 (6.3)	0.093	34	55.8 (4.2)	0.181
Liverpool	60	46.9 (4.8)	0.516	36	44.3 (6.2)	0.361	24	56.4 (6.0)	0.307
Juventus	67	47.2 (4.0)	0.479	45	45.2 (5.3)	0.366	22	51.7 (6.8)	0.810
Bayern München	92	48.7 (3.2)	0.678	54	49.2 (4.0)	0.849	38	52.6 (4.8)	0.597
Barcelona	93	45.1 (3.8)	0.196	54	45.1 (5.4)	0.367	39	61.3 (5.2)	0.043
Olympique Lyon	55	52.5 (4.6)	0.956	39	45.0 (5.3)	0.338	16	60.1 (7.0)	0.175
Internazionale	53	52.5 (4.3)	0.559	39	51.1 (4.8)	0.812	14	60.0 (8.5)	0.274
Arsenal	63	54.4 (4.2)	0.299	42	50.7 (5.5)	0.897	21	62.4 (5.8)	0.047
Porto	69	54.4 (3.2)	0.178	51	49.7 (3.6)	0.921	18	68.0 (5.2)	0.003
Borussia Dortmund	42	54.9 (3.8)	0.211	30	51.8 (4.0)	0.656	12	66.2 (8.1)	0.085
Milan	51	56.3 (5.2)	0.242	33	46.8 (5.6)	0.564	18	74.0 (6.4)	0.004
Atlético Madrid	50	58.9 (4.9)	0.080	33	58.7 (7.1)	0.249	17	60.8 (7.5)	0.180
Roma	38	63.1 (4.1)	0.003	27	59.1 (4.9)	0.078	11	68.9 (5.8)	0.005

AM: Away matches

Table 4 reveals that 30 out of the 42 teams analyzed did not experience a disadvantage when playing away from home in the overall league. Furthermore, Table 6 provides a clear depiction of the variation in AD values among these teams based on the strength of their opponents. For instance, Chelsea, despite not having AD in general, exhibited AD when playing against “strong” teams in the league. However, they did not face a disadvantage when playing against “medium-strong” teams. Interestingly, Chelsea not only avoided a disadvantage, but also gained an advantage when playing against “weak” teams. The findings presented in Tables 4 and 6 underscore the influence of the opponent's strength on the AD values of teams.

**Table 6:** AD (%) for teams against strong, medium-strong and weak teams in the UEFA Champions League.

	all		against “strong” teams		against “medium-strong” teams		against “weak” teams	
Team	Adj (SE)	*p*-value	Adj (SE)	*p*-value	Adj (SE)	*p*-value	Adj (SE)	*p*-value
Paris Saint-Germain	45.1 (3.8)	0.196	61.7 (8.7)	0.223	37.0 (8.2)	0.115	12.5 (19.5)	0.103
Manchester City	45.2 (3.8)	0.207	58.4 (7.3)	0.280	35.2 (5.9)	0.015	36.6 (9.4)	0.159
Real Madrid	46.4 (3.3)	0.276	55.2 (5.0)	0.302	46.8 (6.8)	0.634	24.5 (11.1)	0.034
Manchester United	46.5 (4.0)	0.378	53.0 (5.6)	0.956	42.9 (6.7)	0.287	38.1 (16.8)	0.477
Chelsea	46.7 (3.8)	0.379	59.5 (4.6)	0.050	45.9 (9.4)	0.663	9.7 (6.3)	<0.001
Liverpool	46.9 (4.8)	0.516	58.7 (7.0)	0.238	45.7 (9.1)	0.635	35.4 (16.4)	0.372
Juventus	47.2 (4.0)	0.479	58.5 (6.5)	0.210	42.1 (9.1)	0.388	57.2 (10.8)	0.533
Bayern München	48.7 (3.2)	0.678	56.7 (4.9)	0.187	42.9 (5.4)	0.194	60.9 (25.7)	0.756
Ajax	50.7 (4.5)	0.866	62.1 (8.4)	0.190	41.8 (7.1)	0.254	51.6 (7.2)	0.827
Barcelona	51.6 (3.9)	0.687	59.2 (5.2)	0.090	44.0 (13.3)	0.651	15.8 (12.8)	0.027
Tottenham Hotspur	52.0 (4.9)	0.684	n/a		n/a		n/a	
Sevilla	52.3 (6.4)	0.724	n/a		54.4 (7.9)	0.592	n/a	
Olympique Lyon	52.5 (4.6)	0.956	67.0 (6.2)	0.017	50.0 (6.7)	0.995	13.0 (8.2)	0.001
Internazionale	52.5 (4.3)	0.559	74.4 (94)	0.064	43.2 (7.8)	0.386	54.4 (18.4)	0.827
Valencia	53.4 (5.8)	0.564	65.0 (10.1)	0.204	60.0 (7.7)	0.221	n/a	
Arsenal	54.4 (4.2)	0.299	67.4 (7.4)	0.046	53.9 (6.7)	0.573	29.5 (12.0)	0.100
Porto	54.4 (3.2)	0.178	63.4 (6.0)	0.040	60.5 (6.0)	0.102	29.8 (10.3)	0.060
Borussia Dortmund	54.9 (3.8)	0.211	75.7 (6.4)	0.003	52.4 (6.0)	0.687	n/a	
Villarreal	56.3 (6.7)	0.369	n/a		44.7 (8.1)	0.516	n/a	
Milan	56.3 (5.2)	0.242	67.5 (6.5)	0.021	41.0 (8.0)	0.263	n/a	
Anderlecht	56.3 (8.8)	0.495	n/a		39.2 (12.0)	0.367	n/a	
Club Brugge	56.7 (7.9)	0.417	n/a		64.2 (10.7)	0.251	n/a	
Napoli	56.9 (4.4)	0.128	n/a		53.1 (5.4)	0.567	n/a	
Sporting Lisbon	57.5 (5.9)	0.218	74.4 (8.8)	0.047	n/a		n/a	
Atlético Madrid	58.9 (4.9)	0.080	71.7 (7.7)	0.027	67.6 (10.3)	0.161	10.6 (9.2)	0.004
Monaco	59.6 (5.9)	0.126	n/a		63.2 (7.3)	0.104	n/a	
Schalke 04	59.8 (5.8)	0.112	62.2 (7.5)	0.136	44.1 (8.1)	0.460	n/a	
Olympique Marseille	59.8 (7.0)	0.186	74.0 (9.3)	0.062	60.6 (6.1)	0.107	n/a	
Lille	60.3 (7.1)	0.175	n/a		56.8 (8.3)	0.436	n/a	
CSKA Moscow	60.4 (4.6)	0.033	n/a		61.3 (5.7)	0.066	n/a	
Dinamo Kiev	61.2 (6.1)	0.086	82.0 (8.9)	0.054	59.7 (6.7)	0.173	n/a	
Bayer Leverkusen	61.9 (5.6)	0.047	78.4 (11.0)	0.127	n/a		n/a	
Shakhtar Donetsk	62.0 (4.6)	0.015	61.2 (7.4)	0.164	76.3 (5.5)	<0.001	35.4 (12.4)	0.242
Roma	63.1 (4.1)	0.003	77.4 (6.3)	0.002	56.6 (5.9)	0.273	n/a	
Basel	64.0 (5.4)	0.018	n/a		64.1 (8.8)	0.158	n/a	
Zenit St. Petersburg	65.1 (5.3)	0.011	n/a		72.7 (8.1)	0.033	n/a	
Benfica	65.7 (3.9)	<0.001	62.6 (8.6)	0.186	69.1 (5.3)	0.002	n/a	
Olympiakos	66.5 (4.5)	0.001	70.7 (7.8)	0.034	61.8 (5.8)	0.059	53.8 (13.9)	0.796
Werder Bremen	69.1 (4.4)	<0.001	n/a		74.5 (5.2)	<0.001	n/a	
Galatasaray	69.6 (5.9)	0.006	n/a		63.2 (7.3)	0.104	n/a	
Celtic	71.1 (5.2)	0.001	79.0 (11.7)	0.165	80.4 (4.4)	<0.001	n/a	
PSV Eindhoven	71.9 (4.5)	<0.001	90.5 (5.0)	0.002	64.1 (7.9)	0.114	n/a	

n/a: not applicable

They suggest that certain teams may excel or struggle in away matches based on the caliber of their opponents. Understanding these variations in AD values can assist teams in devising effective strategies and adapting their gameplay when competing against teams of different strengths.

The study found that there was a significant difference $\left(\chi_{28}^{2}=153.9,\;p<0.001\right)$ between 29 teams that played at least 10 away matches against strong opponents in the league. The variation between teams was also observed against “medium-strong” opponents $\left(\chi_{38}^{2}=575.5,\;p<0.001\right)$ and “weak” opponents $\left(\chi_{16}^{2}=1494.8,\;p<0.001\right)$.

When examining AD values at the team level, significant results were obtained, indicating that AD varied depending on the strength of the opponent (*p* < 0.001) for the 17 teams under investigation. Interestingly, it was observed that AD values did not differ significantly when facing “medium-strong” and “weak” opponents (*p* = 0.230). However, significant differences were found in AD values when comparing the same teams' performances against “strong” and “weak” opponents (*p* < 0.001) as well as against “strong” and “medium-strong” opponents (*p* = 0.001). These findings suggest that teams exhibited a lower AD value when facing “weak” opponents, and in some cases, certain teams even demonstrated an advantage when playing against “weak” teams in the league. Moreover, when the “weak” teams were excluded from the comparison, it was observed that teams' AD values significantly varied depending on the opponent's strength (*p* < 0.001) when comparing their performances against “strong” and “medium-strong” teams. These results highlight the dynamic nature of AD values and their dependence on the relative strength of opponents. They suggest that certain teams performed differently in terms of their AD when facing teams of varying strengths. These findings contribute to a deeper understanding of team performance and factors influencing their AD values in different match scenarios.

## Discussion

The purpose of this study was to calculate the advantage of being a home team and the disadvantage of being an away team for teams with a certain number of matches played in the UEFA Champions League. To achieve this goal, the study employed the Generalized Estimating Equations and Poisson Regression methods previously used by Goumas (2017). One advantage of this method is that it provides individualized home advantage estimates for each team, thereby avoiding the influence of other teams' results. Additionally, unlike other methods, it incorporates team abilities into the model. Previous methods tend to “regress” each team's home advantage towards the mean home advantage for all teams combined, which reduces the ability to detect differences among teams.

The traditional HA calculation method (Pollard, 1986) has been utilized in numerous competitions where each team played an equal number of matches against one another (Armatas and Pollard, 2014; Pollard et al., 2008; Pollard and Gomez, 2009, 2014a; Riberio et al., 2022). Also, the amount of competitive balance among the teams in a league has been shown to influence HA when quantified as the percentage of points won by the home team in the study of Pollard and Gómez (2014a). However, using the traditional method in competitions where teams do not play an equal number of matches and compete against teams of varying strengths can be misleading when comparing teams based on these results. For instance, within the dataset used in this study, when considering only matches played against strong teams in the UEFA Champions League, Celtic FC and SSC Napoli yield HA values of 100%. This outcome is a result of both Celtic and Napoli having lost all their matches played against strong teams away. Against this disadvantage of the traditional method, the Generalized Estimating Equations and Poisson Regression methods, based on teams' goal performance, serve as robust alternatives in team-level HA calculations. Moreover, this method's consideration of team abilities for predictions, applicability when teams play varying numbers of matches, provision of statistical reliability for each team, and robustness of outcomes are advantages of this approach. In the measurement of home advantage in soccer, Pollard and Stefani (2021) have summarized and discussed the alternative methods employed.

The results indicate that in the past 20 years of the Champions League, many teams (37 out of 42 teams) with at least 40 matches played had a statistically significant home advantage. However, it was found that this advantage varied significantly (ranging from 32.1% to 79.5%) among teams. These findings differ from those obtained by Goumas (2017) using the same method. The main reason for this discrepancy may be due to the differences in the analyzed teams. In Goumas (2017), all teams except Olympiacos had played in at least one semifinal match (O. Lyon) and reached at least one championship (FC Porto), which is not the case in this study. Nevertheless, the results obtained for the teams included in both studies are consistent. This study examined a larger number of teams and more data, including results from various countries' Champions League teams.

Another contribution of this study is the finding that the home advantage does not significantly differ between the group stage and knockout matches. Furthermore, an important finding regarding the home advantage is its variability depending on the strength of the opponent. It was observed that while all teams had a home advantage against “weak” teams, the advantage decreased and even disappeared for some teams when facing “medium-strong” and “strong” teams. This result, obtained by controlling for the strength of the opponent, distinguishes this study from the others available in the literature that calculated the home advantage without considering the strength of the opponent (Goumas, 2017; Leite and Pollard, 2020; Marek and Vavra, 2017; Matos et al., 2021).

Numerous studies conducted in different countries and leagues have shown that the majority of teams have a home advantage at the team level (Marek and Vavra, 2017; Pollard et al., 2008, 2017; Ramchandani et al., 2021). The main reasons for this phenomenon include crowd support, referees, geographic factors, psychological factors, absence of travel fatigue compared to the opponent, and tactics employed, among many other factors (Almedia and Volossovitch, 2017; Carron et al., 2005; Courneya and Carron, 1992; Pollard, 2008). However, the findings of this study clearly indicate the need for incorporating the strength of the opponent alongside these factors. Analysis of the results demonstrates that the majority of the 42 teams included in the research do not have a home advantage against “strong” opponents. It was only found that certain teams genuinely possessed a home advantage regardless of who the opponent was.

Contrary to the interest in studying the home advantage that teams possess, the literature on the away disadvantage that teams face is scarce (Goumas, 2017). In this study, the teams' away disadvantages were also modeled using the same method. According to the analysis of the results, while most teams had a home advantage, it was found that only 30 teams did not have an away disadvantage. Furthermore, compared to the range of the home advantage (between 32.1% and 79.5%), the range of the away disadvantage (between 45.1% and 71.9%) was smaller. This suggests that being an away team is still an important factor for all teams. Various factors, such as the country visited for the away match, travel conditions and distance, tactics employed, player experience and quality, and other psychological factors, affect away teams to varying extents. Additionally, it was found that the strength of the opponent faced during an away match also influenced the teams' away disadvantage, although this factor did not apply to some teams. Regardless of the opponent's strength, only nine teams (Paris Saint Germain, M. City, Real Madrid, M. United, Liverpool, Juventus, Bayern Munich, Ajax, Barcelona) were found not to experience an away disadvantage. Considering all matches, the result that the advantage possessed by the other 21 teams without an away disadvantage does not apply to every opponent will provide a different dimension to studies on home advantage and away disadvantage. Also, the findings of the research indicate that factors such as spectator pressure, influence on the referee, etc., which are the reasons for home advantage, do not have a significant effect on some teams and their players.

In the literature, there are numerous studies on home advantage (HA) and away disadvantage (AD), most of which focus on specific leagues. The number of studies conducted at the team level is limited, and most of them are related to home advantage. Pollard and Gomez (2009) examined home advantage for teams in the South-West Europe region. In their study, HA values for teams in France ranged from 59.2% to 74.4%, in Italy from 61.0% to 71.8%, in Portugal from 61.0% to 71.3%, and in Spain from 67.1% to 72.0%. Armatas and Pollard (2014) similarly estimated home advantage for individual teams in Greek soccer to range from 49.6% to 80.5%. Pollard et al. (2008) determined that different Brazilian teams had a home advantage ranging from 57.5% to 74.9%. Those studies have shown that while factors such as team quality, crowd size, stadium capacity, and other factors may vary, the majority of teams have a home advantage. In another study that examined home advantage in European leagues (Pollard and Gomez, 2014a), it was found that the average home advantage for males was approximately 60%, with variations ranging from 52.8% to 65.2% across different countries. HA values obtained in this study for teams in the Champions League are in line with the literature. When compared with team-based studies conducted in South-West European countries (Pollard and Gomez, 2009) and Greece (Armatas and Pollard, 2014), very similar results are observed. For example, Marseille has a 68.5% home advantage in Ligue 1, while in this study, it was estimated as 66.2%. Juventus has a 65.4% home advantage in Seria A, while in this study, it was 68.3%. Porto has a 67.4% home advantage in the Portuguese league, while in this study, it was 69.7%. Real Madrid has a 68.9% home advantage in La Liga, while in this study, it was 73.8%. Finally, Olympiacos has a 64.3% home advantage in the Greek Superleague, and it was also estimated as 64.3% in this study. Although different datasets, different time periods, and different methods make direct comparisons of results challenging, the consistency of the findings increases the reliability of the obtained results. However, this study clearly demonstrates that the home advantage possessed by teams is not applicable to every opponent. This finding in the context of the Champions League is likely to be valid for local leagues as well. For example, in La Liga, the majority or all teams have a home advantage, but this advantage is likely limited against teams like Real Madrid or Barcelona. Similar examples can be found in other leagues. Likewise, it is highly plausible that some teams do not have a home advantage against certain opponents. In this regard, the study findings indicate that factors such as the difference in strength and quality between teams, psychological factors among opponents, etc., have a more dominant influence on the outcome of matches than home advantage or away disadvantage. Additionally, comparing the home advantage of teams in European competitions and local leagues using the same methodology holds potential as a future research topic.

The study findings clearly demonstrate the differences among teams participating in the Champions League. For Paris Saint Germain, M. City, Real Madrid, M. United, Liverpool, Juventus, Bayern Munich, Ajax, and Barcelona, the identity of their opponents did not have an impact on their home advantage. Similarly, these teams did not have an away disadvantage against any opponent. The results obtained for these teams, which are among the strongest and have won numerous championships in the Champions League, can contribute to the discussions surrounding the “European Super League” in recent years (Sky Sports, 2021; The Guardian, 2021; The Independent, 2021) in European soccer. Furthermore, these findings can assist in marketing efforts aimed at increasing viewership and generating revenue for the Champions League and local leagues (Holt, 2007; Chadwick and Holt, 2008).

The study has certain limitations, particularly due to the limited amount of data available for some teams. Although the analysis included data from 2,344 matches, the number of teams that played at least 40 matches (20 matches at home, 20 matches away) is only 42. Moreover, there are significant differences in the number of matches among these teams. This situation leads to higher standard error (SE) values for some teams. Similarly, within the analyzed period, only 17 teams had sufficient data in both the group and knockout stages. The finding that the home advantage values remained constant while the away disadvantage values varied is applicable only to these specific teams. Obtaining more data from a larger number of teams would allow for more reliable results to be obtained.

## Conclusions

This study focuses on analyzing the home advantage (HA) and away disadvantage (AD) of teams participating in the UEFA Champions League (CL), which is one of the top-tier European soccer competitions. The findings of the study indicate that among the 42 teams analyzed, 37 teams had a significant HA, while 30 teams did not experience an AD. However, it is noteworthy that only specific teams consistently maintained a HA regardless of the strength of their opponents, and similarly, they did not face a significant AD.

## References

[ref1] Almeida, C. H. & Volossovitch A. (2017). Home advantage in Portuguese football: effects of level of competition and mid-term trends. International Journal of Performance Analysis in Sport, 17(3), 244–255, 10.1080/24748668.2017.1331574.

[ref2] Arboix-Alió, J., Buscà, B., Trabal, G., Aguilera-Castells, & Sánchez-López, M. J. (2020). Comparison of home advantage in men’s and women’s Portuguese roller hockey league. Cuadernos de Psicología del Deporte, 20(1), 181–189.

[ref3] Armatas, V., & Pollard R. (2014). Home advantage in Greek football, European Journal of Sport Science, 14(2), 116–122. 10.1080/17461391.2012.736537. 24533517

[ref4] Carron, A. V., Loughhead T. M. & Bray S. R. (2005). The home advantage in sport competitions: Courneya and Carron's (1992) conceptual framework a decade later, Journal of Sports Sciences, 23(4), 395–407, 10.1080/02640410400021542. 16089184

[ref5] Chadwick, S. & Holt, M. (2008). Building global sports brands: key success factors in marketing the UEFA champions league IN M. Desbordes, Marketing & Football: An international perspective (3rd ed., 21–51). Taylor & Francis Group.

[ref6] Clarke, S. R., & Norman J. M. (1995). Home ground advantage of individual clubs in English soccer. Statistician, 44(4), 509–521.

[ref7] Courneya, K. S., & Carron, A. V. (1992). The home advantage in sport competitions: a literature review. Journal of Sport & Exercise Psychology, 14(1), 13–27.

[ref8] Goumas, C. (2013). Modelling home advantage in sport: a new approach. International Journal of Performance Analysis in Sport, 13(2), 428–439.

[ref9] Goumas, C. (2017) Modelling home advantage for individual teams in UEFA Champions League football. Journal of Sport and Health Science, *6*, 321–326. 30356603 10.1016/j.jshs.2015.12.008 PMC6189255

[ref10] Gryko, K; Mikolajec, K; Marszałek J., Adamczyk J., Molik B., Waśkiewicz Z., Nikolaidis P., & Knechtle, B (2020). How did basketball teams win EuroBasket 2015? A non-standard analysis of performance based on passes, dribbling and turnovers. International Journal of Performance Analysis in Sport, 20 (3), 339-356. 10.1080/24748668.2020.1749013.

[ref11] Harris, A.R., & Roebber P. J. (2019). NBA team home advantage: identifying key factors using an artificial neural network. PLoS ONE, 14(7), e0220630. 31365592 10.1371/journal.pone.0220630 PMC6668839

[ref12] Holt, M. (2007). Global success in sport: the effective marketing and branding of the UEFA Champions League, International Journal of Sports Marketing and Sponsorship, 9(1), 46–56.

[ref13] IBM (2019). IBM Corp. Released 2019. IBM SPSS Statistics for Windows, Version 26.0. Armonk, NY: IBM Corp.

[ref14] Jamieson, J. P. (2010). The home field advantage in athletics: A meta-analysis. Journal of Applied Social Psychology, 40(7), 1819–1848.

[ref15] Leite, W., & Pollard, R. (2020). Comparison of Home Advantage between Level 1 and Level 2 in Women’s Football Leagues. Journal of Anthropology of Sport and Physical Education, 4(4), 9–13.

[ref16] Marek, P. & Vávra F. (2017, June 26–28). Home team advantage in English Premier League. [Paper presentation]. Mathsport International 2017 Conference, Padua, Italy.

[ref17] Matos, R., Monteiro, D., Antunes, R., Mendes, D., Botas, J., Clemente, J., & Amaro, N. (2021). Home-Advantage during COVID-19: An Analysis in Portuguese Football League. International Journal of Environmental Research and Public Health, 18, 3761. 33916536 10.3390/ijerph18073761 PMC8038430

[ref18] Parim, Ç, Güneş, M. Ş., Büyüklü, A. H., & Yıldız, D. (2021). Prediction of match outcomes with multivariate statistical methods for the group stage in the UEFA Champions League, Journal of Human Kinetics, 79, 197–209, 10.2478/hukin-2021-0072. 34400999 PMC8336563

[ref19] Pic, M. (2018). Performance and Home Advantage in Handball. Journal of Human Kinetics, 63, 61–71. 10.2478/hukin-2018-0007. 30279942 PMC6162984

[ref20] Pollard, R. (1986). Home advantage in soccer: A retrospective analysis. Journal of Sports Sciences, 4, 237–248. 2884328 10.1080/02640418608732122

[ref21] Pollard, R., Silva, C. D., & Medeiros, N. C. (2008). Home advantage in football in Brazil: differences between teams and the effects of distance travelled. Revista Brasileira De Futebol (The Brazilian Journal of Soccer Science), 1(1), 3–10.

[ref22] Pollard, R. & Gómez M. A. (2009). Home advantage in football in South-West Europe: Long-term trends, regional variation, and team differences, European Journal of Sport Science, 9(6), 341–352,

[ref23] Pollard, R. & Gómez M. A. (2014a). Comparison of home advantage in men's and women's football leagues in Europe. European Journal of Sport Science, 14(S1), S77–S83, 24444247 10.1080/17461391.2011.651490

[ref24] Pollard, R., & Gómez, M. A. (2014b). Components of home advantage in 157 national soccer leagues worldwide. International Journal of Sport and Exercise Psychology, 12(3), 218–233.

[ref25] Pollard, R., & Gómez, M. Á. (2015). Validity of the established method of quantifying home advantage in soccer. Journal of Human Kinetics, 45(1), 7–8. 25964804 10.1515/hukin-2015-0001 PMC4415821

[ref26] Pollard, R., Armatas V., & Zamanisani S. H. (2017). Home Advantage in Professional Football in Iran–Differences between Teams, Levels of Play and the Effects of Climate, International Journal of Science Culture and Sport, 5(4), 328–339.

[ref27] Pollard, R., Prieto, J., & Gómez, M. Á. (2017). Global differences in home advantage by country, sport and sex. International Journal of Performance Analysis in Sport, 17(4), 586–599.

[ref28] Pollard, R., & Stefani, R. (2021). The Assessment and Quantification of the Home Advantage Effect. In M. A. G. Ruano, R. Pollard & C. L. Penas, Home Advantage in Sport: Causes and the Effect on Performance (31–43). Routledge.

[ref29] Ramchandani, G., Millar, R. & Wilson, D. (2021). The relationship between team ability and home advantage in the English football league system. German Journal of Exercise and Sport Research, 51, 354–361. 10.1007/s12662-021-00721-x PMC8152196 40477580

[ref30] Ribeiro, L. C., Fonseca, F. S., Costa, G. C. T., Castro, H. O., Santos, J. P. V. D. S., & Figueiredo, L. S. (2022). Did the Absence of Crowd Support During the Covid-19 Pandemic Affect the Home Advantage in Brazilian Elite Soccer?. Journal of Human Kinetics, 81, 251–258. 10.2478/hukin-2022-0047. 35291644 PMC8884879

[ref31] Rooney, L. & Kennedy, R. (2018). Home advantage in Gaelic football: the effect of divisional status, season and team ability, International Journal of Performance Analysis in Sport, 18(6), 917–925.

[ref32] European Super League - the key questions: what is it? who is involved? how likely? [Editorial] (18 April 2021). Sky Sports. https://www.skysports.com/football/news/12040/12279788/european-super-league-the-key-questions-what-is-it-who-is-involved-how-likely

[ref33] The Guardian (2021). Harris, Daniel; Ingle, Sean (20 April 2021). "European Super League: backlash builds against breakaway plan – live!". The Guardian. ISSN 0261-3077. Archived from the original on 20 April 2021. Retrieved 20 April 2021. No protection for big clubs in new European Super League proposals [Editorial] (30 July 2021]

[ref34] The Independent (2021). https://www.independent.co.uk/sport/football/european-super-league-premier-league-european-court-of-justice-real-madrid-barcelona-b1894086.html .

[ref35] Van Damme, N., & Stijn, B. (2019). Home advantage in European international soccer: Which dimension of distance matters?, GLO Discussion Paper, No. 314, Global Labor Organization (GLO), Maastricht.

[ref36] Volossovitch, A. & Debanne T. (2021). Home advantage in handball, In Gómez-Ruano M., & Pollard R., & Lago-Peñas C. (Eds.) Home Advantage in Sport: Causes and the Effect on Performance. Routledge. eBook ISBN:9781003081456.

[ref37] Yonghui, Y., Antonio G. A., Kai C., & Tianbiao L. (2020). Interactive effects of home advantage and quality of opponent in Chinese Women’s Volleyball Association League, International Journal of Performance Analysis in Sport, 20(1), 107–117,

